# Broad-Spectrum Antivirals and Antiviral Drug Combinations

**DOI:** 10.3390/v14020301

**Published:** 2022-02-01

**Authors:** Valentyn Oksenych, Denis E. Kainov

**Affiliations:** 1 Institute of Clinical Medicine, University of Oslo, 0318 Oslo, Norway; valentyn.oksenych@ntnu.no; 2 Department for Cancer Research and Molecular Medicine (IKOM), Norwegian University of Science and Technology, 7491 Trondheim, Norway; 3 Institute of Technology, University of Tartu, 50090 Tartu, Estonia

Viral diseases consistently pose a substantial economic and public health burden worldwide. This burden is due to the viruses’ ability to be transmitted from wild and domestic animals to humans, resulting in unpredictable outbreaks [1]. The current strategy for viral outbreak management is heavily reliant on vaccines and antiviral treatments [2]. While the development of novel vaccines is often long, laborious, and unprofitable, broad-spectrum antivirals (BSAs) remain a timely and effective disease management option, which reduce virus transmission from human to human [3]. 

BSAs inhibit the replication of multiple viruses from the same or different viral families. To mitigate the development of antiviral drug resistance and increase their efficacy, antivirals are combined into BSA-containing drug cocktails (BCCs) [4,5,6]. Synergistic BCCs have lower concentrations of antivirals, which reduces their toxicity and side effects. There are hundreds of known BSAs and BCCs [4,7]. 

In this Special Issue, authors report novel, and review known, BSAs and BCCs [8,9,10,11] -. Their results indicate that the landscape of BSAs and BCCs activities and virus coverage is vast, and can be further interrogated and expanded. The basic science data generated by these articles unveil new insights into the interactions between virus and host during viral infections and decipher the mechanisms of action of inhibitors and modulators of these interactions [8,9,10,11,12]. This may help us to uncover critical virus–host interactions and the underlying principles, which determine pan- and cross-family activities of BSAs, as well as understand what makes some BCCs act synergistically, something that is still largely unknown in the medical science community. 

Many more BSAs and BCCs are awaiting discovery and development. However, new methods are needed to identify and prioritize the development of a few of the thousands of potential pan- and cross-virus family therapeutic [5,13]. Such methods can assist the global health community by providing new options to fight ongoing and recurrent viral outbreaks. They can also serve as a general paradigm in the quest for proactive global preparedness for future virus outbreaks by providing cost-effective and life-saving countermeasures that can be deployed during the critical period between virus identification and the development of vaccines, virus-specific drugs, and therapeutic antibodies. 
